# The knowledge, attitude, and practice towards coronavirus disease (COVID-19) among scuba divers in Saudi Arabia: A cross-sectional study

**DOI:** 10.12688/f1000research.73496.3

**Published:** 2026-02-05

**Authors:** Khalifa S. Al-Khalifa, Muneer H. Alshuyukh, Amal Alfaraj, Ashwin C. Shetty, Yaser A. Alsahafi, Abdullah S. Al-Swuailem

**Affiliations:** 1 Department of Preventive Dental Sciences, College of Dentistry, Imam Abdulrahman Bin Faisal University, Dammam, 31441, Saudi Arabia; 2 College of Dentistry, Imam Abdulrahman Bin Faisal University, Dammam, 31441, Saudi Arabia; 3 Department of Prosthodontics and Dental Implantology, College of Dentistry, King Faisal University, Al-Ahsa, Saudi Arabia; 4 Department of Dental Education, College of Dentistry, Imam Abdulrahman Bin Faisal University, Dammam, 31441, Saudi Arabia; 5 Department of Community Dentistry and Oral Epidemiology, College of Dentistry, Qassim University, Buraidah, Saudi Arabia; 6 Department of Periodontics and Community Dentistry, College of Dentistry, King Saud University, Riyadh, 11545, Saudi Arabia

**Keywords:** SARS-CoV-2, Recreational divers, Pandemic, Infection control, Perception, Saudi Arabia

## Abstract

**Background:**

The aim of this study was to investigate the knowledge, attitude, and practice (KAP) among scuba divers in Saudi Arabia towards equipment infection control measures, protective guidelines measures and potential post COVID-19 infection complications.

**Methods:**

A cross-sectional study using a pre-validated questionnaire was conducted. The questionnaire consisted of 35 close-ended questions, which covered the scuba divers’ profile and experience, the KAP of COVID-19 precautionary measures during diving activities as well as the demographic data and background of the scuba divers. KAP scores were subjected to non-parametric tests of statistical significance (Mann Whitney and Kruskal Wallis test). Statistical significance was set at p < 0.05.

**Results:**

Most of the 461 participants’ answers were in agreeance on the proper KAP of infection control during the COVID-19 pandemic. There was a statistically significant difference in attitude between all the demographic and professional variables (p<0.05) except for gender and region of residence (p>0.05). In addition, there was a statistically significant practice difference among age groups, education levels, and employee status in the diving center (p<0.05).

**Conclusions:**

Our findings showed that scuba divers presented a good level of KAP of infection control measures against the spread of the coronavirus disease. Local officials and diving organizations need to continue their efforts in combating and control the spread of this pandemic.

## Introduction

Coronavirus disease (COVID-19) is caused by a new RNA, zoonotic virus, known as severe acute respiratory syndrome coronavirus 2 (SARS-CoV-2). ^1^ SARS-CoV-2 is a highly contagious virus that was first recognized as the cause of extremely infectious pulmonary disease and pneumonia cases in the city of Wuhan, China causing an epidemic across China and subsequently spreading in other countries all over the world. ^1^ In March 2020, the World Health Organization (WHO) recognized the COVID-19 disease outbreak as a pandemic disease. ^2^ COVID-19 symptoms may vary from mild to severe clinical presentations. ^3^^,^^4^ As declared by the American Centers for Disease Control and Prevention (CDC), the most common symptoms include fever, dry cough, fatigue, and dyspnea which may appear after 2-14 days after exposure to the virus. ^3^^,^^4^ The first case of COVID-19 in Saudi Arabia was confirmand on Monday 2 March 2020. ^5^ Similar to other countries, the Saudi government has developed solid guidelines to prevent the virus from spreading among the population. ^5^

The COVID-19 pandemic has greatly affected all aspects of life worldwide. Social distancing measures, lockdown of businesses, schools and overall social life have been strictly applied to prevent the spread of the disease, and this has certainly impacted many life routines and practices including sports and physical activities. ^5^^,^^6^ Scuba diving was one of the sport activities that was greatly disrupted worldwide because of the pandemic and, as with many other activities, updated guidelines were implemented to restart diving activities while placing emphasis on the safety of the diving community. ^6^^,^^7^ To overcome the consequences of the pandemic, the Saudi Arabian government (similar to other countries) called for a gradual return to normal life activities with strict adherence to global public health guidelines to prevent the spread of the disease and protect the public. ^5^

On Monday 1 June 2020, the General Directorate of Borders Guards, Ministry of Interior officially permitted the practice of recreational Scuba diving in Saudi Arabia. ^8^ Moreover, international diving agencies such as the Professional Association of Diving Instructors (PADI), ^9^ National Association of Underwater Instructors (NAUI), ^10^ and Scuba Schools International (SSI) ^11^ with the partnership of Divers Alert Network Europe (DAN) ^12^ developed new health and safety guidelines to allow safe sea diving. Physical distancing which is considered one of the most important preventive measures against the spread of COVID-19 was not a common practice in the pre- COVID-19 era where divers are required to check their partners’ equipment for safety purposes before diving. Moreover, due to limited space and a low number of divers/area ratio on local boats, any attempts at social distancing might be compromised. The use of rented diving gear such as diving masks, snorkels, regulators, and buoyancy control devices (BCD) among divers may result in the spread of infection if this diving gear is not properly disinfected. In addition, the Belgian Society for Diving and Hyperbaric Medicine (SBMHS-BVOOG) ^13^ indicated that divers who have recovered from COVID-19 infection may temporarily or permanently lose the medical fitness to dive. Therefore, symptomatic or asymptomatic divers who had COVID-19 infection might be at higher risk of complications during diving. ^13^ For instance, lung barotrauma has a higher chance of occurring during uncontrolled ascent if there is already damage to the lung due to COVID-19 infection. ^13^^,^^14^ In addition, cardiac arrest and decompression sickness (DCS), which are potentially fatal events, have a higher chance of occurring after COVID-19 infection recovery. ^13^

The risk associated with diving during the COVID-19 pandemic is undoubtedly high. Therefore, it is important to know a diver’s knowledge, attitude, and commitment to health guidelines and protective measures during the COVID-19 pandemic. Previous studies have shown that adequate knowledge and appropriate preventive practices toward COVID-19 are critical for infection control and public safety. For instance, research among nursing students has demonstrated that gaps in knowledge and attitudes may adversely affect compliance with preventive measures, highlighting the need for targeted educational interventions. ^15^ However, data on knowledge, attitude, and practices among the diving community to prevent the spread of COVID-19 is lacking in the literature. Therefore, this study aimed to investigate the knowledge, attitude, and practice (KAP) among scuba divers in Saudi Arabia towards equipment infection control measures, protective guidelines measures and potential post COVID-19 infection complications.

## Methods

### Ethics statement

The study was reviewed and approved by the ethical committee of the Imam Abdulrahman Bin Faisal University (ethical approval number EA 202159). The participants were informed that their participation in this study was for research purposes and public health awareness, and that the data associated with the study would be published. Participants were informed that their consent to participate was assumed when they agreed to fill the questionnaire. Study participants’ agreement to be part of the study was reflected in their positive response to the study invitation. The implied consent to participate was deemed sufficient by the institutional review board upon granting ethical approval.

### Study design

This cross-sectional study was conducted to assess KAP among scuba divers in different provinces of Saudi Arabia regarding the COVID-19 pandemic and its impact on Scuba diving sport. The study outcome was the KAP among scuba divers in Saudi Arabia towards equipment infection control measures, protective guidelines measures and potential post-COVID-19 infection complications. Demographic information and diving experience were considered study predictors.

### Survey tools

After permission was sought from the authors of the Blue Atlantic Project questionnaire for its reuse, their pre-validated questionnaire ^16, 17^ was reviewed and several questions regarding diving profile and demographic data were added. The amended version of the questionnaire was created upon the recommendations of the Scuba diving international agencies DAN and PADI on the best practices to reduce COVID-19 transmission risk. ^9– 12^ In addition, the updated questionnaire utilized new recommendations concerning resuming Scuba diving post-COVID-19 infection posted by the Belgian Society for Diving and Hyperbaric Medicine (SBMHS-BVOOG). ^13^

The questionnaire comprises 35 close-ended questions, divided into five sections. The first section included five questions about the scuba divers’ profile and experience. The second section comprised of five questions about the knowledge of COVID-19 precautionary measures during diving activities. The participants had to choose from three options: “yes,” “no,” and “I do not know”. The third section consisted of seven questions about the attitude of the scuba divers toward COVID-19 precautionary measures while diving. They had to choose from three options: “Agree,” “Neutral,” and “Disagree”. The fourth section included eleven questions which covered the practice of COVID-19 precautions while diving. Divers had to choose from the option of “Yes” and “No”. The fifth and final section covered seven questions on the demographical data and background of scuba divers. A copy of the survey tool is available in *Extended data*. ^30^

### Survey piloting

The questionnaire was pilot tested on 20 randomly selected local Saudi Arabian scuba divers. Invitations to participate in the pilot test were sent via WhatsApp to one scuba diver WhatsApp group in Dammam, Saudi Arabia. The participants in the pilot test were not part of the main study group. The questionnaire was originally structured in English and then translated to Arabic and then back-translated to English to ensure proper translation by the authors of the study. The Arabic questionnaire was tested on 10 divers with different diving experience levels. In addition, the English version of the questionnaire was pilot tested on 10 English-speaking divers. The participants were given identification codes and were asked to fill the questionnaire twice (to compare answers) within 2-6 days. Feedback was taken from these divers via WhatsApp, after which some questions were rephrased, and some questions were updated according to the feedback. This helped in modifying the questionnaire for better readability and validity.

### Setting

The study employed a web-based questionnaire constructed via Google Forms software which were eventually sent via seven private scuba diving WhatsApp groups in Saudi Arabia which contained hundreds of scuba divers’ phone numbers. The authors approached the owners of WhatsApp groups in order to distribute the questionnaire within their groups. The questionnaire was distributed between 6 October 2020 to 5 December 2020. A follow up reminder was sent to the study participants one week and two weeks after the initial invitations were sent.

### Participants

The sample size was calculated considering a 95% confidence level, 5% margin of error. As per the latest statistics from the Saudi Federation of Water Sports and Diving, ^18^ the number of scuba divers in the country was expected to be approximately 10,000 divers. Based on the 50% response distribution, the minimum required sample size was calculated using Raosoft online sample size calculator to be 370. The random sample shared common demographic characteristics. The study included any recreational divers in Saudi Arabia who confirmed they were active scuba divers and willing to answer the survey. Of the 2000 individuals invited to participate, 461 completed the online questionnaire, resulting in a response rate of 23.05%.

### Data coding

The sum score of each outcome was assessed based on Bloom’s cut-off point. ^19^ Participants’ knowledge questions had a value of 1 or 0 (correct response had a value of “1” and wrong or don’t know response had a value of “0”). The score for each of the five knowledge questions would range from 0 to 5 points. Participants’ overall knowledge was classified into low-level knowledge [0,3], moderate-level knowledge, ^3, 4^ and high-level knowledge. ^4^^,^^5^

Similarly, seven questions related to attitude were graded on a 3-point Likert scale, an agreement scale ranging from ‘1’ for disagree to ‘3’ for agree. The overall level of attitude was categorized as positive attitude [80% - 100%], neutral attitude [60% - 80%], and negative attitude (less than 60%). Subsequently, level of practice was classified into poor level (less than 60%), fair level (60% - 80%), and good level (80% - 100%).

### Data analysis

The data was entered in Microsoft Excel (2010) and transferred to IBM SPSS Statistics for Windows, version 22 (IBM Corp., Armonk, NY, USA) for statistical analysis.

In the case of missing data, the complete entry for the study subject was removed. Descriptive statistics included frequency distributions with percentages for different demographic variables such as age, gender, education, region of residence, employment status, and the status of employment in the diving center. Along with this, the distribution of chronic diseases reported among scuba divers was illustrated.

Frequency distributions with percentages were also calculated for scuba divers’ profile and their experience.

Questions about the knowledge, practice, and attitude of scuba divers towards the COVID-19 pandemic was represented in bar graphs as percentages.

KAP scores were subjected to non-parametric tests of statistical significance (Mann Whitney and Kruskal Wallis test). Statistical significance was set at p ≤ 0.05.

## Results

### Univariate analysis

The survey was sent to 2000 recreational divers in different WhatsApp groups in Saudi Arabia, 461 responses were returned, indicating a response rate of 23.05%. Table 1 represents the demographic and professional characteristics of sampled scuba divers. Of the total 461 participants, approximately two-third were below 40 years old (n = 304, 66.0%). Most of the respondents were male (n = 416, 90.2%), had University level education (n = 284, 61.6%), were employed (n = 341, 74.0%), and were residing in the eastern region of Saudi Arabia (n = 269, 58.4%). Only 15.4% (n = 71) were employed in a diving center. A total of 70 participants (15.2%) reported chronic diseases.

**Table 1.** Demographic and professional characteristics of participants.

Variable	Description	Frequency (Percent) n (%)
Age in years	18-29	122 (26.5)
	30-39	182 (39.5)
	≥ 40	157 (34.1)
Gender	Male	416 (90.2)
	Female	45 (9.8)
Education	High school education and below	78 (16.9)
	Institution/Academy education	99 (21.5)
	University education	284 (61.6)
Employment status	Unemployed	70 (15.2)
	Self-employed	50 (10.8)
	Employed	341 (74.0)
Region of residence	Eastern	269 (58.4)
	Western	125 (27.1)
	Others	67 (14.5)
Employed in diving center	Yes	71 (15.4)
	No	390 (84.6)

The majority of the participants reported having PADI (n = 403, 87.45) as their scuba diving certification agency and owned specialized and basic dive gear (BCD, Regulator) (n = 298, 64.6%). The distribution of the responses on the current level of diving experience, years of diving, and source of knowledge about COVID-19 precautionary measures with diving activities is shown in Table 2.

**Table 2.** Scuba divers’ profile and experience. BCD, buoyancy control device.

Variable	Description	n (%)
What is your scuba diving certification agency?	PADI	403 (87.4)
	NAUI	26 (5.6)
	Others	32 (6.0)
What is your current level of diving experience?	Beginner (open water diver)	103 (22.3)
	Intermediate (advanced-adventure open water diver)	120 (26.0)
	Advanced (rescue OR dive master diver)	124 (26.9)
	Instructor	114 (24.7)
How many years have you been diving?	< 1 year	54 (11.7)
	1-2 years	99 (21.5)
	3-5 years	112 (24.3)
	6-10 years	86 (18.7)
	More than 10 years	110 (23.9)
Do you own your personal dive gear?	No	22 (4.8)
	Basic Dive Gear (Mask, snorkel, Fins)	141 (30.6)
	Specialized and basic dive gear (BCD, Regulator)	298 (64.6)
I gained my knowledge about COVID-19 precautionary measures with diving activities from:	My diving agency	131 (28.4)
	Saudi federation of water sports & diving	39 (8.5)
	My diving center & instructor	132 (28.6)
	I did not get any information	159 (34.5)

**Figure 1.**
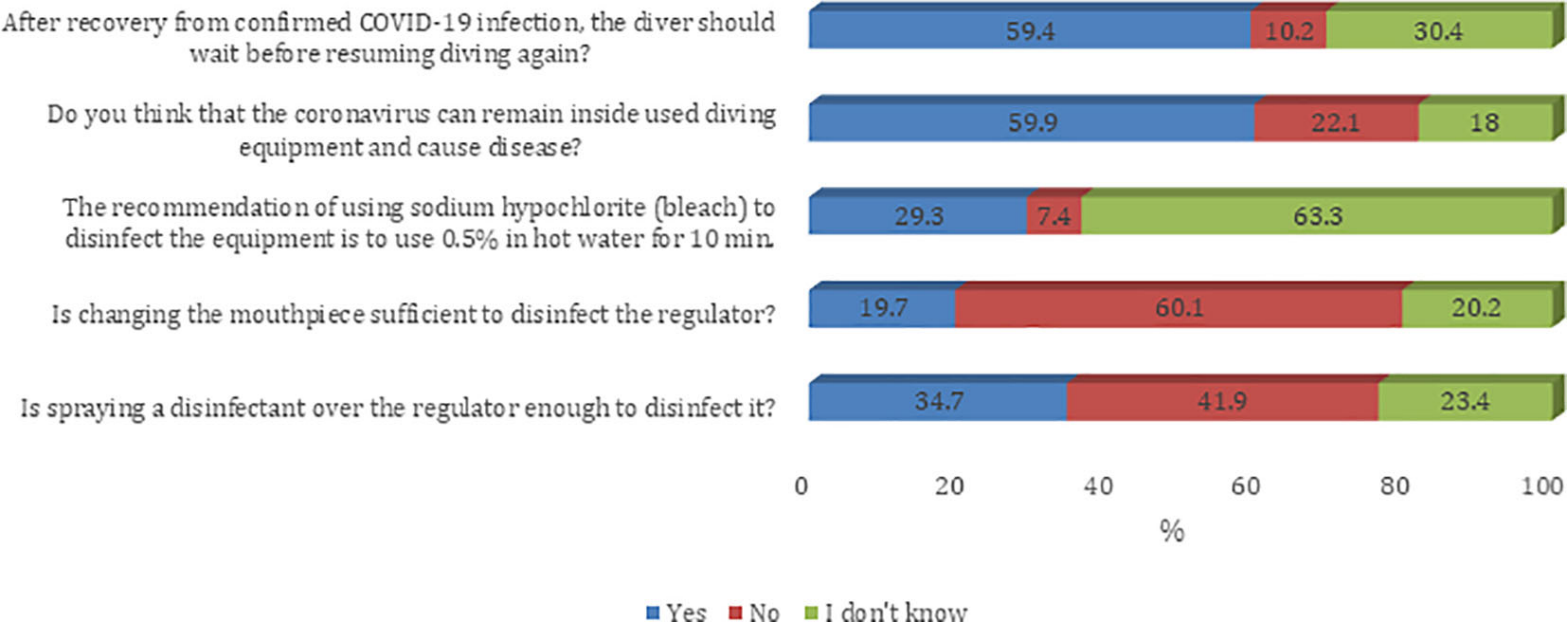
Participants’ knowledge about infection control measures against the COVID-19 pandemic.

**Figure 2.**
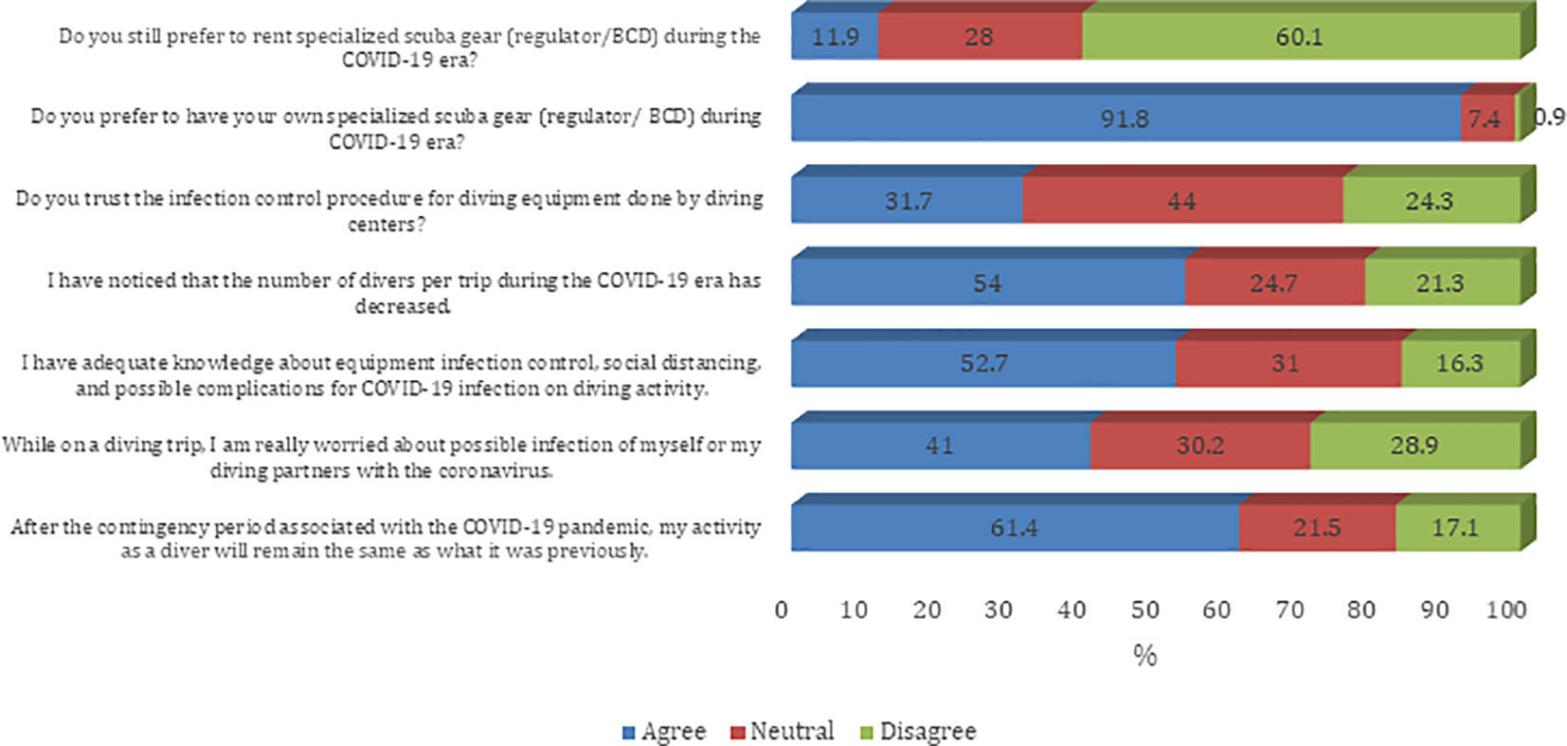
Participants’ attitude towards infection control measures against the COVID-19 pandemic. BCD, buoyancy control device.

**Figure 3.**
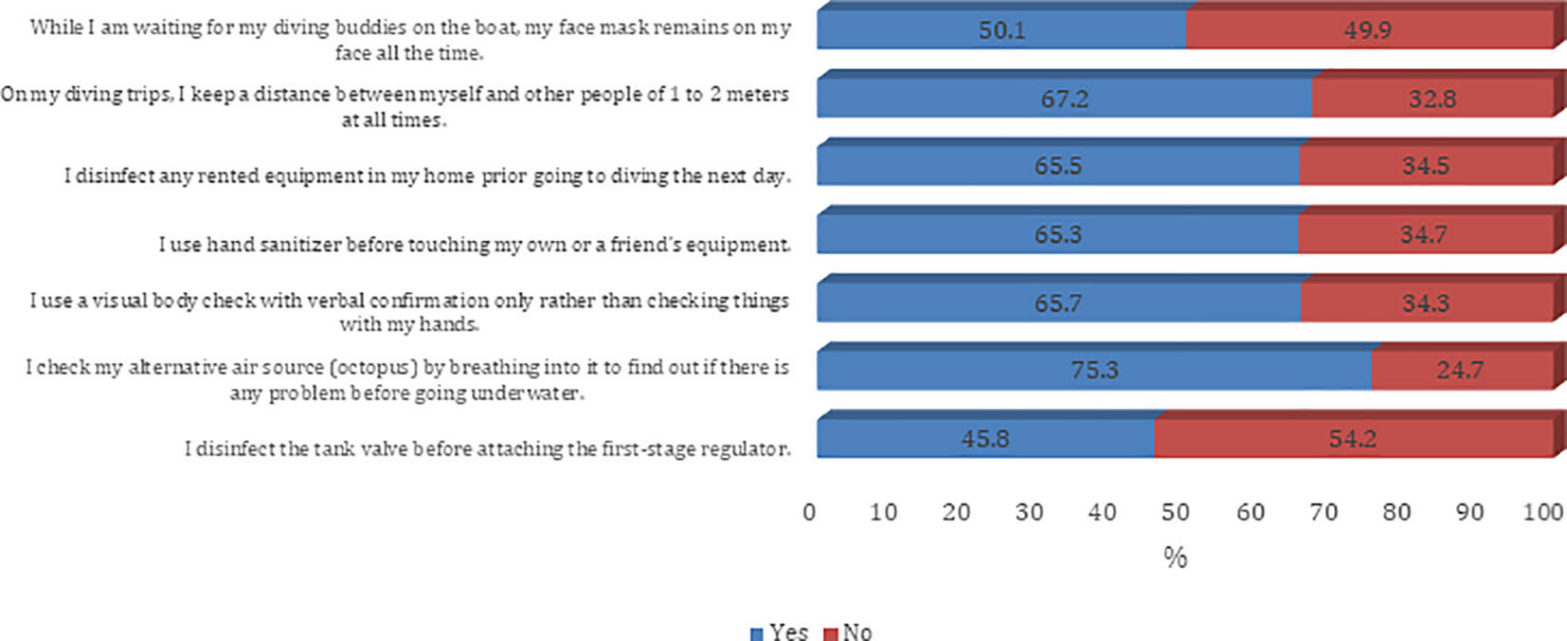
Participants’ practice around infection control measures against the COVID-19 pandemic.

**Figure 4.**
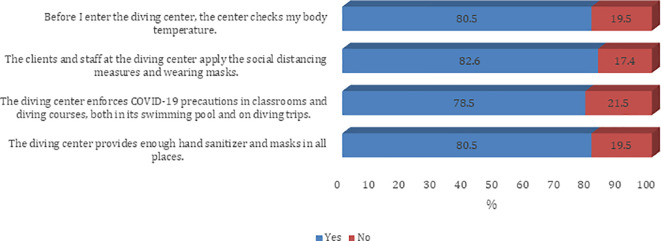
Participants’ practice about infection control measures against the COVID-19 pandemic (Dive center).

Most of the participants agreed on questions about the knowledge, attitude, and practice towards the COVID-19 pandemic. The majority of sampled divers (59.4%) believe that after recovery from confirmed COVID-19 infection the diver should wait before resuming diving again. In addition, 59.9% agree that the coronavirus can remain inside used diving equipment and can cause COVID-19 infection ( Figure 1). Most of the participants agree that they prefer to have their own specialized scuba gear (regulator/BCD) during the COVID-19 era (91.8%). Furthermore, most of the participants believe that their diving activity will remain the same as what it was before the COVID-19 pandemic (61.4%). Also, sampled divers reported a decreased number of divers per trip during the COVID-19 era (54.0%). About half of sampled divers believe that they have adequate knowledge about equipment infection control, social distancing, and possible complications for COVID-19 infection on diving activity (52.7%) ( Figure 2). The majority of the participants agree on all questions on procedures and guidelines to prevent the spread of COVID-19 infection except the need to disinfect the tank valve before attaching the first-stage regulator (45.8%) ( Figures 3 and 4).

### Bivariate analysis

Overall, 54.5% of participants had low-level knowledge, 24.5% had moderate-level knowledge, and 21.2% had high-level knowledge. Female participants had moderate-level knowledge compared to male participants with low level of knowledge. However, there was no statistically significant difference in the knowledge (p > 0.05) ( Table 3).

**Table 3.** Comparison of knowledge score by demographic and professional characteristics.

Variable	Description	Median (IQR)	Mean rank	p value
Age in years	18-29	2.0 (2.0)	211.8	0.057
	30-39	2.0 (2.0)	228.4	
	≥ 40	2.0 (2.0)	24.0	
Gender	Male	2.0 (2.0)	228.8	0.273
	Female	3.0 (1.5)	251.2	
Education	High school education and below	2.0 (1.0)	243.2	0.227
	Institution/Academy education	2.0 (2.0)	212.0	
	University education	2.0 (2.0)	234.5	
Employment status	Unemployed	2.0 (2.0)	222.6	0.700
	Self-employed	2.0 (2.0)	222.0	
	Employed	2.0 (2.0)	234.0	
Region of residence	Eastern	2.0 (2.0)	226.2	0.510
	Western	2.0 (2.0)	242.4	
	Others	2.0 (2.0)	229.0	
Employed in diving center	Yes	2.0 (2.0)	254.2	0.103
	No	2.0 (2.0)	226.8	

Overall, 53.2% of participants had a negative attitude, 45.7% had a neutral attitude, and only 1.1% had a positive attitude. There was a statistically significant attitude difference between all the demographic and professional variables (p < 0.05) except gender and region of residence (p > 0.05). Participants aged 18-29 years who were female, university educated, unemployed, and from the western region had a neutral attitude. All the other groups had negative attitudes ( Table 4).

**Table 4.** Comparison of attitude score by demographic and professional characteristics.

Variable	Description	Median (IQR)	Mean rank	p value
Age in years	18-29	13.0 (3.0)	257.49	0.018*
	30-39	12.0 (2.0)	228.93	
	≥ 40	12.0 (2.0)	212.81	
Gender	Male	12.0 (2.0)	229.47	0.447
	Female	13.0 (2.0)	245.17	
Education	High school education and below	11.0 (3.0)	170.67	0.000*
	Institution/Academy education	12.0 (3.0)	198.74	
	University education	13.0 (3.0)	258.82	
Employment status	Unemployed	13.0 (3.0)	264.31	0.044*
	Self-employed	12.0 (3.0)	241.78	
	Employed	12.0 (2.0)	222.58	
Region of residence	Eastern	12.0 (3.0)	231.68	0.704
	Western	13.0 (2.0)	235.79	
	Others	12.0 (3.0)	219.32	
Employed in diving center	Yes	12.0 (3.0)	196.80	0.017*
	No	12.0 (2.0)	237.23	

Overall, 55.8% had poor level practice, 19.1% had a fair level, and 25.1% had good level practice. There was a statistically significant practice difference among age groups, education levels, and employment status in diving centers (p < 0.05). Participants aged ≥ 40 years, with institution/academy and high school education or less, who were self-employed, who had ‘others’ as region of residence, and who were employees in diving centers had fair level practice. All the other groups had poor level practice ( Table 5).

**Table 5.** Comparison of practice score by demographic and professional characteristics.

Variable	Description	Median (IQR)	Mean rank	p value
Age in years	18-29	3.0 (3.0)	191.05	0.000*
	30-39	4.0 (2.0)	232.09	
	≥ 40	5.0 (3.0)	260.78	
Gender	Male	4.0 (3.0)	230.0	0.621
	Female	4.0 (4.0)	240.0	
Education	High school education and below	5.0 (3.0)	265.10	0.000*
	Institution/Academy education	5.0 (2.0)	272.49	
	University education	4.0 (3.0)	207.17	
Employment status	Unemployed	4.0 (3.0)	205.32	0.193
	Self-employed	5.0 (3.25)	242.06	
	Employed	4.0 (4.0)	234.65	
Region of Residence	Eastern	4.0 (4.0)	225.73	0.200
	Western	4.0 (2.0)	228.14	
	Others	5.0 (2.0)	257.49	
Employed in diving center	Yes	5.0 (2.0)	299.86	0.000*
	No	4.0 (3.0)	218.46	

Overall, all (100.0%) had good level practice. There was a statistically significant difference in following the recommended guidelines to prevent the spread of COVID-19 infection among different age groups and education levels (p < 0.05) ( Table 6). The survey responses are provided in *Underlying data*. ^29^

**Table 6.** Comparison of practice score by demographic and professional characteristics (dive centers).

Variable	Description	Median (IQR)	Mean rank	p value
Age in years	18-29	4.0 (2.0)	210.16	0.039*
	30-39	4.0 (1.0)	232.94	
	≥ 40	4.0 (1.0)	244.95	
Gender	Male	4.0 (1.0)	232.02	0.557
	Female	4.0 (1.0)	221.53	
Education	High school education and below	4.0 (1.0)	248.16	0.000*
	Institution/Academy education	4.0 (0.0)	264.72	
	University education	4.0 (2.0)	214.53	
Employment status	Unemployed	4.0 (1.0)	222.15	0.333
	Self-employed	4.0 (1.0)	242.25	
	Employed	4.0 (1.0)	231.17	
Region of residence	Eastern	4.0 (1.0)	236.58	0.249
	Western	4.0 (1.0)	216.53	
	Others	4.0 (1.0)	235.58	

## Discussion

After the resumption of recreational scuba diving activities during the COVID-19 pandemic, several safety guidelines and precautions have been released to guide the divers. ^20^ However, there is insufficient information about the awareness among divers regarding safety protocols and post COVID-19 infection complications. This study is unique and the first to be reported among scuba divers. Results of this study will form a foundation for the studies to follow in these specific professions and other similar professions.

As the COVID-19 infection primarily affects the lungs, it has implications for the health of scuba divers. It is important that recovered COVID-19 divers must wait and attain fitness before resuming diving after their recovery. ^20^ In this research, participants’ responses suggest that most of the divers were aware of the waiting period before resuming diving after recovery from the COVID-19 infection.

A study by Yalin *et al*. suggests that the use of a regulator to breathe may aid in the transmission of the virus. ^21^ So, disinfecting regulators by submerging in a disinfectant solution is recommended. In this study, more than half of the divers were aware of the increased chances of coronavirus transmission from used diving equipment. The WHO recommends the use of sodium hypochlorite as a surface disinfectant for 10-60 minutes to kill coronavirus from the equipment surface. ^22^ However, only a few of the respondents were aware of the method to disinfect the diving equipment using 0.1% sodium hypochlorite in cold water for a minimum of five minutes. ^22^^,^^23^ Most of the respondents believed that changing the mouthpiece of the regulator or spraying a disinfectant was not sufficient to disinfect the regulator.

Studies suggest that coronavirus can breed easily in diving equipment that are worn out or have developed cracks. ^21^ Therefore, it is best to avoid renting the equipment for scuba diving, in light of the pandemic. In our study, most respondents preferred to have their own specialized scuba gear, with more than half of scuba divers preferring not to rent specialized scuba gear. The participants also reported a lack of trust in the infection-control measures for equipment being followed at scuba diving centers. There was also lesser participation among divers due to the pandemic, with more than half of divers reporting less than the usual number of divers. The lower participation is likely to be driven by awareness about possible complications of COVID-19 infection. ^21^ Many divers reported being worried about themselves and their diving partners getting infected with the coronavirus. However, there was optimism about the future after the pandemic, with ~60% of respondents believing that their activity as divers will remain the same as the previous after the pandemic.

In addition to awareness about the possible complications and exposure to themselves and their scuba diving partners, a high share of respondents also reported high awareness about safety protocols to be followed. However, the divers did not prefer keeping their face masks on at all times, possibly due to physical discomfort and inconvenience caused by the face masks. ^24^ About half of the divers said they do not prefer keeping a mask on the face while waiting for their diving buddy. In terms of using social distancing as a precautionary measure, most respondents reported maintaining a distance of 1-2 meters. Among other measures, respondents said they were disinfecting their rented equipment before using the next day, and preferred using a hand sanitizer before using their own or others’ equipment. The majority of them had a visual body check with verbal confirmation and also made use of alternative air sources by breathing into it before going underwater. The whirl of testing the alternative air source by breathing into it for checking technical issues before dive is not recommended anymore for the duration of the pandemic. The virus can remain inside the hoses of the regulator and may infect the diver who will need it in emergency situations. ^9^ However, many divers (54.2 %) reported that they do not disinfect the tank valve before attaching the first stage regulator.

Precautions being followed at the diving centers were largely reported to be adequate. When asked about infection control measures against the COVID-19 pandemic at the diving center, 4 in 5 respondents said they experienced temperature checks before entering the diving centers. About four out of five respondents also felt that clients and staff at the diving center practiced adequate social distancing norms and were wearing face masks. About 78% of divers felt that diving centers were enforcing COVID-19 precautions in classrooms and diving courses, both in swimming pools and during diving trips. Most of them felt that the diving center provided a sufficient amount of sanitizer and an adequate number of face masks at all locations.

In the present study, scuba divers showed a good level of practice of infection control measures. These results are similar to the level of the general Saudi population where knowledge, attitude, and practices towards COVID-19 were 81.3%, 86.6%, and 81.9%, respectively. ^25^ Similarly, Alhanawi *et al*. also reported that the mean COVID-19 knowledge score was 17.96, indicating a high level of knowledge. ^5^ Our study has typically pointed out each profession had shown more responsibility by understanding the infectious disease and displayed the knowledge which is essential at this emergency time of infection control. Scuba divers need to be more careful about the infection control measures because their equipment and accessories need to be followed with stringent infection control measures.

In the present study, female participants had a moderate level of knowledge compared to male participants with low level of knowledge. However, there was no statistically significant difference in the knowledge. One of the studies among the general population showed that men have less knowledge, less optimistic attitudes, and less good practice toward COVID-19 than women. ^5^ Other studies found that older, female, and more educated respondents are more knowledgeable about emerging communicable diseases. ^26^^,^^27^

A positive attitude during a contagious infection is always important to contain the infection. ^5^ In the present study, there was a statistically significant attitude difference between all the demographic and professional variables except gender and region of residence. Participants aged 18-29 years, who were female, university educated, unemployed, and from the western region of residence had a neutral attitude. All the other groups had negative attitudes. This result is similar to previous reports. Alhanawi *et al*. showed a positive and optimistic attitude toward COVID-19. ^5^ In their study, approximately 94% showed that the virus can be successfully controlled and 97% agreed that the Saudi government will control the pandemic. This type of attitude is possible only because of the right action taken at right time by the government, such as lockdown at the right time and suspension of all transport measures where there was a possible transmission. This was also reported in a similar study in China where most of the subjects agreed that the disease is curable and that their country will combat the disease. ^28^

### Limitations and recommendations

This study has several limitations. Its cross-sectional design limits causal inference and does not capture changes in knowledge, attitudes, or practices over time as COVID-19 guidelines evolved. The use of a self-administered, web-based questionnaire may have introduced reporting and social desirability bias. Recruitment through WhatsApp groups may have resulted in selection bias toward more engaged or informed divers, and the relatively low response rate limits the generalisability of the findings to all recreational scuba divers in Saudi Arabia. In addition, the rapidly changing scientific evidence and public health recommendations during the study period may have influenced participants’ responses. Furthermore, the use of convenience sampling and the observed response rate may limit the generalizability of the findings. Finally, reported practices may not fully reflect actual behaviors.

Despite these limitations, the findings provide practical implications for policy and practice. Policymakers and relevant authorities should develop and regularly update standardised national guidelines for scuba diving during infectious disease outbreaks, with specific emphasis on equipment disinfection, group management, and post-infection medical fitness to dive. Regulatory bodies should consider implementing routine monitoring and certification of dive centres to ensure compliance with these guidelines. Diving agencies and centres are encouraged to provide mandatory, structured training on infection control measures for both staff and divers. End users, particularly recreational divers, should be targeted through clear risk-communication strategies that emphasise personal responsibility, correct equipment hygiene, and adherence to medical clearance recommendations after COVID-19 infection. Future studies using longitudinal designs and more representative sampling are recommended to evaluate behavioural changes over time and the impact of implemented policies. For end users, adherence to evidence-based preventive guidelines and participation in ongoing training are essential to reduce infection risk and protect public health.

### Public health implications

This study is important to public health because it highlights how well scuba divers understand and apply infection control measures in a high-risk recreational setting involving shared equipment and close contact. By identifying gaps in knowledge, attitudes, and practices related to COVID-19, the findings can inform targeted health education, safer diving guidelines, and policy decisions to reduce disease transmission and prevent diving-related complications, thereby protecting both the diving community and the wider public.

From a public health perspective, understanding knowledge, attitudes, and practices related to COVID-19 among scuba divers is important, as this group represents a future component of the healthcare workforce. Identifying knowledge gaps and suboptimal practices can inform targeted public health education, improve adherence to infection prevention measures, and strengthen preparedness for future infectious disease outbreaks.

## Conclusions

Our findings showed that scuba divers presented a good level of knowledge, attitude, and practice of infection control measures against the spread of the coronavirus. Most of the dive centers have followed proper practice against the spread of the coronavirus as reported by the divers. The KAP of infection control measures against the spread of the coronavirus will continue to be updated as a better understanding of the novel COVID-19 is gained. Local officials and diving organizations need to continue in the efforts to combat and control the spread of this pandemic.

## Data Availability

Harvard Dataverse: The Knowledge, Attitude, and Practice Towards Coronavirus Disease (COVID-19) Among Scuba Divers in Saudi Arabia. https://doi.org/10.7910/DVN/4VX3GP. ^29^ This project contains the following underlying data:
- Scuba data raw Version 2.tab (survey responses). Data are available under the terms of the Creative Commons Zero “No rights reserved” data waiver (CC0 1.0 Public domain dedication). Figshare: The knowledge, attitude, and practice towards coronavirus disease (COVID-19) among scuba divers in Saudi Arabia. https://doi.org/10.6084/m9.figshare.16866508.v1. ^30^ This project contains the following extended data:
- Scuba survey.docx (questionnaire). Data are available under the terms of the Creative Commons Attribution 4.0 International license (CC-BY 4.0).
