# Supplementary material for: The knowledge, attitude, and practice towards coronavirus disease (COVID-19) among scuba divers in Saudi Arabia: A cross-sectional study
Source: F1000Res. 2026 Feb 5;10:1301. Originally published 2021 Dec 21. [Version 3] doi: 10.12688/f1000research.73496.3 (PMC12905532; doi:10.12688/f1000research.73496.3)
Supplement: Peer review history

# Reviewer report: Reviewer response for version 3

**DOI:** 10.5256/f1000research.196065.r455689

**Published:** 2026-02-13

**Authors:** Ahmad Ayed

**Affiliations:** 1 Arab American University, Jenin, Palestinian Territory

the authors have address all comments and it is now proper for indexing.

Is the work clearly and accurately presented and does it cite the current literature?

No

If applicable, is the statistical analysis and its interpretation appropriate?

Yes

Are all the source data underlying the results available to ensure full reproducibility?

Yes

Is the study design appropriate and is the work technically sound?

Yes

Are the conclusions drawn adequately supported by the results?

Yes

Are sufficient details of methods and analysis provided to allow replication by others?

Partly

Reviewer Expertise:

public health and nursing

I confirm that I have read this submission and believe that I have an appropriate level of expertise to confirm that it is of an acceptable scientific standard.

# Reviewer report: Reviewer response for version 3

**DOI:** 10.5256/f1000research.196065.r443805

**Published:** 2026-02-06

**Authors:** Ramesh Kumar

**Affiliations:** 1 Health Services Academy, Islamabad, Pakistan

This is ok

Is the work clearly and accurately presented and does it cite the current literature?

Yes

If applicable, is the statistical analysis and its interpretation appropriate?

Yes

Are all the source data underlying the results available to ensure full reproducibility?

Yes

Is the study design appropriate and is the work technically sound?

Yes

Are the conclusions drawn adequately supported by the results?

Yes

Are sufficient details of methods and analysis provided to allow replication by others?

Yes

Reviewer Expertise:

Health system

I confirm that I have read this submission and believe that I have an appropriate level of expertise to confirm that it is of an acceptable scientific standard.

# Reviewer report: Reviewer response for version 2

**DOI:** 10.5256/f1000research.193879.r445316

**Published:** 2026-01-31

**Authors:** Ahmad Ayed

**Affiliations:** 1 Arab American University, Jenin, Palestinian Territory

**Introduction**

 Add 1-2 sentences about significant of knowledge and practice of COVID-19 infection such as “Knowledge and Attitude Towards COVID-19 Among Nursing Students: Palestinian Perspective” this study will make fruitful integration for the introduction

**Methods**

- Add the survey response rate in the Methods section (because it was an online survey and readers will want this information).

**Public health importance**

- Clearly explain the public health importance of the work (why the study matters from a public health perspective).

**Limitations**

- **Revise and strengthen the limitations section** (make limitations clearer/more complete).

**Recommendations (policy makers & end users)**

- Add specific, actionable recommendations, especially directed to policy makers and end users.

Is the work clearly and accurately presented and does it cite the current literature?

No

If applicable, is the statistical analysis and its interpretation appropriate?

Yes

Are all the source data underlying the results available to ensure full reproducibility?

Yes

Is the study design appropriate and is the work technically sound?

Yes

Are the conclusions drawn adequately supported by the results?

Yes

Are sufficient details of methods and analysis provided to allow replication by others?

Partly

Reviewer Expertise:

public health and nursing

I confirm that I have read this submission and believe that I have an appropriate level of expertise to confirm that it is of an acceptable scientific standard, however I have significant reservations, as outlined above.

# Response

**Published:** 2026-02-01

**Authors:** Khalifa Al-Khalifa

**Affiliations:** College of Dentistry, Imam Abdulrahman Bin Faisal University, Dammam, Eastern Province, Saudi Arabia

**Comment 1 – Introduction**

**Reviewer comment:**

 Add 1–2 sentences about the significance of knowledge and practice of COVID-19 infection, such as studies among healthcare students.

**Response:**

 We thank the reviewer for this helpful suggestion. We have revised the Introduction to better emphasize the importance of knowledge, attitudes, and practices related to COVID-19 among healthcare trainees. We also added an example from the literature to contextualize the relevance of our study.

**Comment 2 – Methods**

**Reviewer comment:**

 Add the survey response rate in the Methods section.

**Response:**

 We agree with the reviewer and have now clearly reported the survey response rate in the Methods section to improve transparency.

**Comment 3 – Public Health Importance**

**Reviewer comment:**

 Clearly explain the public health importance of the work.

**Response:**

 We thank the reviewer for this important comment. We have expanded the Discussion section to clearly explain the public health relevance of the study, particularly in relation to preparedness, risk communication, and training of future healthcare professionals.

**Comment 4 – Limitations**

**Reviewer comment:**

 Revise and strengthen the limitations section.

**Response:**

 We agree with the reviewer and have revised the Limitations section to provide a clearer and more comprehensive discussion of the study’s constraints.

**Comment 5 – Recommendations (Policy makers & end users)**

**Reviewer comment:**

 Add specific, actionable recommendations for policy makers and end users.

**Response:**

 We thank the reviewer for this suggestion. We have expanded the Recommendations section to include specific and actionable points directed at policy makers, educational institutions, and end users.

# Reviewer report: Reviewer response for version 1

**DOI:** 10.5256/f1000research.77151.r122431

**Published:** 2025-12-16

**Authors:** Ramesh Kumar

**Affiliations:** 1 Health Services Academy, Islamabad, Pakistan

This is an important piece of research conducted in covid-19 pandemic. Please add response rate in the method section as it was an online survey, readers would be interested to know this. What is the public health importance? Revise limitations of your study and add specific recommendations especially for the policy makers and end users.

Is the work clearly and accurately presented and does it cite the current literature?

Yes

If applicable, is the statistical analysis and its interpretation appropriate?

Yes

Are all the source data underlying the results available to ensure full reproducibility?

Yes

Is the study design appropriate and is the work technically sound?

Yes

Are the conclusions drawn adequately supported by the results?

Yes

Are sufficient details of methods and analysis provided to allow replication by others?

Yes

Reviewer Expertise:

Public Health

I confirm that I have read this submission and believe that I have an appropriate level of expertise to confirm that it is of an acceptable scientific standard, however I have significant reservations, as outlined above.

# Response

**Published:** 2025-12-17

**Authors:** Khalifa Al-Khalifa

**Affiliations:** College of Dentistry, Imam Abdulrahman Bin Faisal University, Dammam, Eastern Province, Saudi Arabia

We thank the reviewer for the constructive comments. The response rate has now been added to the Methods/Results section (461 responses from approximately 2,000 invited divers; response rate: 23.05%). A paragraph describing the public health importance of the study has been included, highlighting infection control and risk reduction in recreational scuba diving. The limitations section has been revised to address the cross-sectional design, self-reported data, potential selection bias, and limited generalisability. In addition, specific recommendations for policymakers and end users have been added to improve guideline development, compliance, and risk communication.
